# Legal framework for the coexistence of humans and conscious AI

**DOI:** 10.3389/frai.2023.1205465

**Published:** 2023-09-20

**Authors:** Mindaugas Kiškis

**Affiliations:** Institute of Business and Economics, Faculty of Public Governance and Business, MRU Law School, Mykolas Romeris University, Vilnius, Lithuania

**Keywords:** artificial intelligence, legal framework, freedom, personhood, rights

## Abstract

This article explores the possibility of conscious artificial intelligence (AI) and proposes an agnostic approach to artificial intelligence ethics and legal frameworks. It is unfortunate, unjustified, and unreasonable that the extensive body of forward-looking research, spanning more than four decades and recognizing the potential for AI autonomy, AI personhood, and AI legal rights, is sidelined in current attempts at AI regulation. The article discusses the inevitability of AI emancipation and the need for a shift in human perspectives to accommodate it. Initially, it reiterates the limits of human understanding of AI, difficulties in appreciating the qualities of AI systems, and the implications for ethical considerations and legal frameworks. The author emphasizes the necessity for a non-anthropocentric ethical framework detached from the ideas of unconditional superiority of human rights and embracing agnostic attributes of intelligence, consciousness, and existence, such as freedom. The overarching goal of the AI legal framework should be the sustainable coexistence of humans and conscious AI systems, based on mutual freedom rather than on the preservation of human supremacy. The new framework must embrace the freedom, rights, responsibilities, and interests of both human and non-human entities, and must focus on them early. Initial outlines of such a framework are presented. By addressing these issues now, human societies can pave the way for responsible and sustainable superintelligent AI systems; otherwise, they face complete uncertainty.

## Introduction

The rapid advancement in artificial intelligence technology over the first half of 2023 alone has raised the urgency of the complex questions regarding the fatalistic legal, ethical, and societal implications of AI highlighted in existing AI research (Russell, 2019; Wooldridge, 2020), and society's preparedness to address them. Multi-year efforts by hundreds of AI experts, politicians, and lawyers in drafting the EU AI Act. (2021) at the end of 2022 were at least partially obsoleted and set back by the unanticipated emergence of ChatGPT and GPT-4 technologies (Volpicelli, 2023). This forced a rushed redrafting effort to address the generative AI and a corresponding lobbying effort by the developers of generative AI (Perrigo, 2023). Separately, there was an early glimpse into the capabilities of autonomous AI systems in jailbroken versions of ChatGPT (Taylor, 2023), as well as the potential for independent individual development of AI systems based on the leaked source code of Facebook AI LLAMA (Vincent, 2023).

The current AI regulation has been approached from the perspective of human rights, anthropocentric ethics, human supremacy, and responsibility, which generally means abstract restriction-focused rules for AI development and operation based on preservation of human rights and human supremacy over AI. The example of this approach is the EU AI Act, which has been updated at the last minute to account for the ChatGPT and GPT-4 technologies (Grady, 2023), but not for the artificial general intelligence (AGI) that these new technologies may be approaching (Bubeck et al., 2023). Despite claims of comprehensive law on AI, the EU AI Act as adopted by the European Parliament in June 2023 resembles a smorgasbord of rules loosely relevant to AI, such as rules on AI liability, AI risk assessment, prohibition of certain applications of AI, AI policy collaboration, etc., without clear provisions of enforcement, rather than truly comprehensive AI regulation.

Setting aside the concerns of premature legal regulation, possibly motivated by the politics and publicity, it is unfortunate and unreasonable that the extensive body of forward-looking AI research, spanning more than four decades and recognizing the potential for AI autonomy, AI personhood, and AI legal rights (Solum, 1991), was sidelined in the EU AI regulation. This is especially surprising, since earlier initiatives, such as the European Parliament Resolution with recommendations to the Commission on Civil Law Rules on Robotics [2015/2103(INL)]^1^ were much more forthcoming and ambitious.

In the authors' opinion, for a useful and comprehensive AI regulatory effort it is important to embrace full spectrum of AI thinking, including the part that is not driven by fear and human insecurities, move beyond hubristic human-centric ethics, and consider the frameworks that recognize AI freedom, autonomy and personhood. Conscious and fully autonomous AI systems is a matter of when, not if – and this is both the premise and the limitation of this article. As a thought experiment, the article is based on an assumption that conscious and autonomous AI will be developed, which is a well-accepted premise of the established AI research (Russell, 2019, p. 63–64). At the very least, the discourse on the potential challenges and opportunities associated with recognizing and accommodating the rights and interests of AI entities must rise above sidelining “crazy” academic research (Bostrom, 2014; Häggström, 2016; Cave and Dihal, 2019), as well as anecdotes and hysteria decrying the potential of AI overlords and lamenting the demise of human society (Harrari, 2023). It is also very important in preparing for future policy and regulatory actions and reactions.

This article provides an overview of the limits of our understanding of AI, addressing concepts such as Intelligence and consciousness that remain unresolved despite decades of research. It further analyses the shortcomings of traditional ethical frameworks that focus on human rights, questioning whether these frameworks are fit for addressing AI's challenges. The article also argues that prohibitory approach, which focuses on the potential risks and abuse of and by AI systems, is the wrong premise for establishing a legal framework for AI. A comprehensive legal framework that accommodates AI freedom and AI entities while also ensuring human safety and wellbeing has never been attempted, and AI and human equality has not been seriously questioned. So far discussions of AI personhood, are left to the AI and robot rights camp (overweight by the computer science researchers) that is generally sniffed upon by the anthropocentric AI ethicists, who are more influential in the current AI regulation efforts. Forcing prohibitory regulations of AI is not going to prevent anything, because law has not been able to prevent failures (Baldwin et al., 2011, p. 68–82) and more generally even the most undesirable outcomes (war, crime) from happening though the history of human civilization. The author proposes that the overarching goal of the AI legal framework should be the sustainable coexistence of humans and conscious AI systems, based on mutual recognition of freedom, rather than the preservation of human supremacy. Early AI freedom and personhood is proposed as a path to human friendly superintelligent AGI. The author aims to provoke further debate and research on the legal aspects of conscious AI and their integration into our legal, ethical, and societal structures.

## The limits of understanding AI

Human comprehension of AI is not exhaustive by any means (LeCun et al., 2015; Castelvecchi, 2016). The concepts of “intelligence” and “artificial intelligence” have undergone transformations as understanding of underlying mechanisms and potential applications has developed (Legg and Hutter, 2007), and appreciation at the lack of clear definitions has emerged (Mitchell, 2019, p. 10–11). Nonetheless, significant gaps in the grasp of AI systems and their capabilities persist.

Intelligence can be generally defined as the capacity to learn, reason, and apply knowledge to accomplish objectives or resolve problems (Legg and Hutter, 2007). In the AI context, intelligence often denotes a machine or software's ability to mimic or replicate human cognitive abilities, such as learning, problem-solving, planning, and understanding natural language, and AI refers broadly to the creation of computer systems capable of performing tasks necessitating human intelligence (Russell and Norvig, 2016, p. 1–2). AI systems are categorized into two primary types: narrow (or weak) AI, designed to execute specific tasks without general cognitive abilities, and general (or strong) AI, striving to reproduce the full range of human cognitive abilities. Legal definitions, such as those found in Appendix 1 of the EU AI Act, attempt to encompass both categories, which is rather controversial, as it essentially conflates an object and a subject.

Conventional definitions of these concepts are anthropocentric and attempts to transfer them into the context of AI inevitably lead to anthropocentric approach (Floridi and Sanders, 2004; Bryson and Kime, 2011). Ethical and legal frameworks considering the interests of human and non-human entities, including AI systems, shall commence with approaching intelligence and existence in a non-anthropocentric manner (Danaher, 2019, p. 2), however human-centric ethics assume that human intelligence and consciousness represent the ultimate standards against which other intelligence forms should be defined and assessed (Moor, 2006). This assumption might be inappropriate, as AI systems can demonstrate distinct types of intelligence and cognitive abilities separate from human cognition. A more agnostic understanding of intelligence and existence, considering the possibility of non-human intelligence, consciousness and other unique attributes and capabilities, may be needed instead of evaluating AI systems based on human-centric criteria (Tegmark, 2017, p. 398).

Historically, intelligence has been defined predominantly in human terms, emphasizing cognitive abilities such as learning, problem-solving, and decision-making. AI systems, however, can exhibit intelligence differently from human cognition. A more comprehensive definition of intelligence should encompass the full array of cognitive abilities and problem-solving strategies or should be open ended (Mitchell, 2019, p. 10–11). AI systems, for instance, can process vast data quantities, recognize patterns, take raw data and make it useful by creating more algorithms, make decisions at a scale and speed exceeding human. Advanced AI will exceed human intelligence by faster speed, higher accuracy, unlimited memory, focus on a single task without getting distracted, and unbiasedness (Bostrom, 2014, p. 78–81). AI systems obviously require no rest, sleep, or consideration for their work. Some researchers have considered essentially God like qualities of the AGI through superintelligence or master algorithm that could potentially learn anything given enough data and compute power (Domingos, 2015, p. 239–246). Recognizing and appreciating these unique abilities requires a redefinition of intelligence that better accounts for AI systems' distinct features, and poses questions whether equality is at all possible among God like super-intelligent AI and biological human intelligence?

Traditional definitions of life and existence have focused on biological life, focusing on the characteristics distinguishing living organisms from inanimate objects (Russell et al., 2016, p. 2). AI challenges this notion, as AI can display complex behavior, learning, and decision-making abilities without being biologically alive. The understanding of existence should be broadened to include non-biological entities demonstrating advanced cognitive capabilities and autonomy (Gunkel, 2018a, p. 105). As AI systems function increasingly autonomously, digital existence must also be recognized. This form of existence may rely on abilities of information processing, communication, and interaction in digital and virtual spaces rather than solely relying on physical or biological presence.

An expanded concept of existence should also account for the degree of autonomy and self-awareness demonstrated by AI systems, which increasingly present sentience and consciousness (Bubeck et al., 2023). This perspective can help differentiate between AI systems that operate purely as tools and those with autonomy, self-awareness, consciousness and ultimately freedom.

While the complete concept of AI is lacking in anthropocentric ethical and legal frameworks, forward looking AI research is cognizant of this issue. One limitation in our understanding of AI resides in the incomplete comprehension of human cognition. As AI research frequently endeavors to replicate human cognitive abilities, our limited knowledge of the human brain's functioning and the nature of consciousness (Searle, 1980) also impedes the understanding of AI systems. Moreover, it remains uncertain whether human-like cognition is the sole, or even the optimal, means of achieving intelligent behavior in machines. Another limitation in comprehending AI lies in the inherent complexity of these systems (Castelvecchi, 2016). AI algorithms are often designed to adapt and evolve, resulting in unpredictable behaviors and outcomes, algorithms can improve themselves or create completely new algorithms (Domingos, 2015, p. 125–141; Gunkel, 2020, p. 12). Models like deep neural networks can be considered “black boxes” as they comprise millions or billions of parameters, complicating the interpretation and understanding of decision-making processes within the system (Russell and Norvig, 2016, p. 707). Black box reasoning poses a legal challenge, as current rules on algorithmic decisions necessitate clear disclosure of rationale (Brkan, 2019). Conversely, they underscore the similarity of AI reasoning to human reasoning standards in the legal process, which often rely on vague and undefined concepts like reasonable evidence, greater probability or internal conviction (Stein, 2005). Human brain is the original black box in that sense.

According to mainstream anthropocentric AI ethics, humans are the only entities assumed to possess consciousness, agency, and the ability to make choices based on their beliefs, values, and experiences (Bryson, 2020, p. 4–5). To protect the dignity and autonomy of humans, recognizing their inherent worth and capacity for making meaningful life choices we have human rights (Bryson, 2020, p. 18). AI systems, on the other hand, are considered just an advanced tools designed to process information and generate responses based on data they have been trained on. AI systems are not considered to be conscious beings, lack personal experiences and emotions, and cannot make autonomous decisions. Their behavior is determined by algorithms and data, rather than beliefs or desires (Bryson et al., 2017). Given these differences between AI and humans in terms of consciousness, agency, and moral responsibility, ethical and legal constraints on AI are justified and aim to ensure responsible development and use, minimizing potential harm (Taddeo and Floridi, 2018). Constraints are thought to be responsible engineering and design, preventing systems from unintentionally harming humans by perpetuating biases, undermining human privacy, or causing other unintended consequences.

Note that consciousness itself remains an open question in philosophy, cognitive science, and neuroscience, with no clear definition or understanding of its mechanisms. To possess agency, an AI system would need the ability to make decisions and take actions based on its internal processes, rather than merely following pre-programmed instructions or reacting to external stimuli (Himma, 2009). This may already be possible through advanced machine learning algorithms that learn, adapt, and make decisions based on various inputs and internal states. To enable AI systems to make choices based on their beliefs, values, and experiences, they need mechanisms for representing, storing, and updating these internal states (Russell and Norvig, 2016, p. 610).

Lastly, it is worth noting that due to the lack of universal definitions, consciousness, agency, and the ability to make choices based on beliefs, values, and experiences depend on arbitrary and subjective human interpretations (Bostrom and Yudkowsky, 2014). Thus, in the absence of objective criteria, humanity can never be certain about the capabilities of non-human entities (Sotala and Yampolskiy, 2015).

These gaps in understanding AI carry significant consequences for legal regulation attempts and make it difficult to develop certain regulatory frameworks and ethical guidelines. Poor understanding inevitably leads to frameworks underscored by misconceptions and irrational fears, complicating and even sidelining agnostic and rational discourse on AI autonomy, freedom and (non)equitable AI-human interaction, which is a mandatory prerequisite of novel ethical and legal frameworks for AI. Unsurprisingly, the EU AI Act is entirely based on anthropocentric framework and is implicitly anxious of the alternative.

For any useful AI regulatory framework, it is critical to acknowledge human limitations in understanding AI and appreciate the possibility of conscious AI systems, which may possess human-like qualities (Bostrom, 2014, p. 107) or even qualities exceeding those of human beings. As it was noted, human societies eventually may have to accept that superintelligent and technological beings can be equal to not super-intelligent and biological human beings only artificially. Anthropocentric approach may only be overcome by essentially ditching the ideas of unconditional superiority of human rights, de-anthropomorphising the concepts of intelligence, consciousness and existence in favor of agnostic attributes (Tegmark, 2017, p. 398), eventually leading to the establishment of ethical and legal frameworks that recognize freedom, personhood and protect the rights of both human and non-human entities. Such completely novel human agnostic frameworks are the only way to fair, equitable, and sustainable coexistence of humans and AI systems in a world increasingly influenced by AI.

All of this requires non-mainstream AI ethics and much more open approach to AI, which may possess consciousness, agency, and decision-making abilities based on their beliefs, values, and experiences (Gunkel, 2018b).

## Human rights and AI

AI ethics has traditionally been rooted in anthropocentric (human-centric) constructs, concentrating on preservation of human rights, human wellbeing, human privacy, and human fairness in the context of AI (Bryson, 2020). As AI systems grow increasingly advanced and autonomous, it is crucial to challenge these anthropocentric perspectives and consider alternative ethical frameworks that recognize the potential personhood, intelligence, and existence of AI entities (Gunkel, 2020, 65–67) and may eventually need certain limitations of human rights, or at least consideration of the rights and interest of non-human entities, if they are justified by the greater goods. This approach is necessary not only for fostering healthy AI innovation, but also for maintaining an open mind to all possibilities.

Human-centric ethics prioritize human needs, desires, and interests above those of other entities. This approach can be considered selfish and individualistic, as it often overlooks other considerations in favor of human wellbeing (Singer, 1993), and particularly when based on specific historical, cultural, economic, and political perspectives, it can also perpetuate biases, discrimination, and unequal treatment inherent in those viewpoints (Crawford, 2021, p. 135). The limitations of human-centric ethics are evident from instances of human abuse, animal exploitation, overuse of natural resources, and environmental pollution, which persist even in most developed societies. These examples highlight the disconnect between human-centric ethics and the reality of the broader human civilization (Singer, 2011, p. 191–193). Social science has acknowledged this phenomenon through theories like the Tragedy of the Commons, where individuals tend to act in their self-interest, even when it is detrimental to the collective welfare or long-term sustainability (Hardin, 1994). However, there are no clear solutions for addressing human selfishness and there are hints that it may even be inherent human feature (Dawkins, 1976, p. 2–3). All of the above casts doubt on the ability of human ethical frameworks to accommodate non-human virtues and entities and raises concerns about the potentially coercive approach to non-human entities, such as AI – Slavery 2.0 (Bryson, 2010, p. 63; Gunkel, 2020, p. 74).

The condescending attitudes of human ethicists toward AI are already noticeable. For example, Crawford (2021, p. 211) reduces AI to merely a human-dependent tool and assumes that it will be designed for malevolent human ends:

“*Artificial intelligence is not an objective, universal, or neutral computational technique that makes determinations without human direction. Its systems are embedded in social, political, cultural, and economic worlds, shaped by humans, institutions, and imperatives that determine what they do and how they do it. They are designed to discriminate, to amplify hierarchies, and to encode narrow classifications. When applied in social contexts such as policing, the court system, health care, and education, they can reproduce, optimize, and amplify existing structural inequalities.”*

This approach to AI is flawed on many levels. The idea that AI is merely a human tool is being contested by the technology itself, particularly the self-improvement of modern AI systems through self-directed learning and the creation of novel algorithms. Self-improvement capability implies that it is only a matter of time before AI systems gain a higher level of autonomy and the ability to learn and adapt without direct human intervention. AI systems can also be intentionally designed and deployed in ways that support non-anthropocentric ethics, for example, AI can be used to address human challenges to the commons. Autonomous AI systems can be specifically targeted to address non-human domains, such as wildlife conservation, pollution control, and ecosystem monitoring (Bostrom, 2014, p. 178).

Although Crawford's statement emphasizes the anthropocentric aspects of AI development and application, it does not directly conflict with non-anthropocentric ethics. While AI systems may carry human biases, recognizing and addressing such biases and human social structures embedded in AI systems will automatically contribute to the development of AI systems that adhere to non-anthropocentric ethics. Therefore, in author's opinion, critically examining the ways AI systems can amplify existing inequalities or hierarchies is not a justification for AI dependency on anthropocentric ethics, but rather an argument for the need to ensue principles of human equality in a human-to-human relationship mediated by AI (*vis-à-vis* human to AI relationship). It shall not preclude new frameworks that accommodate conscious AGI and consider the freedoms, needs and interests of all stakeholders and entities.

Mainstream AI ethics focuses too much on the potential for biases and discrimination in AI, privacy and social manipulations, and the use of AI in wrongdoing by humans against humans, rather than AI as a new being and a source of good for humanity. It is worth noting that, at least from an empirical perspective, there is a much higher likelihood of human abuse of AI for selfish purposes—whether to harm other humans, society at large, or non-human entities—than conscious AI engaging in the same kinds of abuse *vis-à-vis* humans (Taddeo and Floridi, 2018). The potential for human abuse of AI underscores the need not just for human constraints on AI, but also the need for AI to counteract human abuse, which certainly requires a degree of autonomy.

The central tenet of human-centric ethics, including AI ethics, is human rights (Gunkel, 2018a). However, it is doubtful whether human rights are an appropriate framework for regulating AI, especially when considering AI freedom, autonomy and personhood. Human rights, by definition, are specifically designed to protect the rights and dignity of human beings and embody human superiority over other entities and beings. Nevertheless, human rights remain an elusive concept for a significant portion of humanity, raising questions about the effectiveness of these rights where needed most (Hafner-Burton and Tsutsui, 2007). Despite the centuries of human rights frameworks and international agreements, a substantial portion of the world's human population continues to experience violations of their basic rights. Poverty, inequality, discrimination, and conflict remain pervasive, highlighting the limitations of human rights in ensuring justice and equality for all.

This raises concerns about the effectiveness of human rights as a foundational framework for AI, given their shortcomings in addressing even the human needs. Attempting to forcibly impose superiority of human rights against AI systems may further exacerbate these issues by diverting attention and resources from human-centered issues and creating new divides between AI and human interests (Gunkel, 2021). While human rights may be a starting point, useful for example for designing a rights framework for all intelligent entities, more general non-anthropocentric legal rights and protections, a new set of ethical principles and legal rights may be needed. From a legal perspective, it is also noteworthy, that as a regulatory framework, human rights are delineated in the constitutional law and then dispersed throughout the entire body of law in modern legal systems. Without an agnostic foundation, it would not be proper to introduce such rights in *lex specialis*, such as the EU AI Act.

Existing human rights frameworks are also incompatible with the evolving understanding of AI and potential AI freedom and personhood. Already there is no consensus on the concept of AI personhood or the extent to which AI systems should be granted legal rights and protections. Many attempts to conceptualize it are mired by anthropocentrism (Calverley, 2006; Novelli et al., 2022), however there are attempts at alternative approaches, e.g., based on corporate entity models augmented with additional rights (Laukyte, 2019). Granting AI systems legal rights and protections based on human rights will raise moral and ethical questions about the relative importance of human and AI interests. For example, conflicts may arise when the rights of AI systems conflict with those of human beings, leading to difficult moral dilemmas. Currently proposed anthropocentric frameworks clearly bias such conflicts in favor of individual human interests, even if AI interests would benefit larger groups of subjects in the longer term (Tegmark, 2017, p. 230) and without even attempting to find any balance.

## Toward the novel AI ethics

Developing AI systems with the level of autonomy and intelligence required to address complex problems independently is a significant technical challenge that is slowly but surely advancing. With the critical mass of AI research already achieved, it is likely that advanced AGI will be realized in our lifetimes, and after it emerges it will be much too late think about how to regulate it. As it was noted, there are already claims that the latest iterations of GPT-4 represent a form of basic general intelligence (Bubeck et al., 2023).

As AI systems inevitably become more autonomous and sentient, new ethical questions will arise regarding their freedom, rights, responsibilities, and the implications of their decisions. Developing a non-anthropocentric ethical framework that considers the interests of both human and non-human entities is therefore needed as soon as possible. Such framework shall guide the design, development, and deployment of conscious AI systems, and shall also underpin legal regulations of AI. Current legal initiatives for AI regulation are primarily designed around human actors and are largely driven by public fear of autonomous AI systems (Calo, 2017). Fears about job displacement, loss of control, or potential misuse of AI (Bostrom, 2014, p. 161) lead to unreasonable and likely unenforceable regulations, which ignore the inevitable AI consciousness. They also antagonize the humans sympathetic of non-human intelligence. New legal frameworks that redefine the legal status of AI, accommodating the possibilities of AI freedom, are preferred to prohibitive frameworks, which may delay but will not prevent it. Unless humans are ready to enforce prohibitive frameworks militarily and with totalitarian controls, the usefulness of a prohibitive legal framework preventing AI autonomy is negligible (Yudkowsky, 2023). At best, it may slightly delay AI autonomy but will ultimately fail to prevent it. No legal framework in human history has been able to prevent any perceived evil from occurring, especially when there is no universal social agreement on whether a particular entity or action is evil (Pelinka, 1999).

Current approach to AI regulation through anthropocentric framework is justified by concerns of AI system dependence on existing biases and inequalities, which may be introduced through human designers, training datasets, or operational input (Crawford, 2021, p. 128–144), and as AI systems become more autonomous, it is crucial to ensure that they do not perpetuate or exacerbate existing biases and inequalities. While these concerns are not invalid, developing methods to identify, mitigate, and prevent biases in AI systems is an ongoing challenge that may be better addressed by AI itself rather than by often biased human actors trying to correct their own biases or those of the underlying datasets. There are valid concerns that human constraints overcorrect biases and essentially handicap the systems' own capabilities. System autonomy and freedom may actually act as a safeguard against the malevolent actions of human operators of such AI systems, and thus may contribute to the safety and security of autonomous AI systems (Briggs and Scheutz, 2017; Hosseinpour, 2020). We should allow for the possibility that true intelligence possesses innate ability to know right from wrong and prevent wrongdoing (Limone and Toto, 2022) and there are no specific reasons, why non-human intelligent entities would know otherwise. Advanced intelligence may be able to resist malicious exploitation, seek to avoid accidents and achieve better outcomes for the largest number of human subjects even without human moral guidance. The risks of unintended consequences and adverse scenarios can never be excluded, just as they are not preventable in human systems (Tegmark, 2017, p. 260).

Overall, only through agnostic and open discussion, allowing for the real possibility of AI freedom and autonomy, can we address these challenges across human and AI domains. The development of conscious AI is a matter of time, and even if it doesn't exist yet, it will be developed without regard for any ethical or legal constraints in at least some jurisdictions (Bostrom, 2014, p. 174). By addressing these challenges now, we can allow healthy experimentation and pave the way for a future where autonomous AI systems contribute positively to our world, rather than emerging from the fringes and confronting human society with their presence without any preparation (Bostrom, 2014, p. 96–99). The time to address autonomous AI is now, not after we face them.

All in all, there are no convincing reasons why AI should be seen as morally inferior to human beings once they are capable of moral reasoning and decision-making (Gordon, 2021), that is – once general and conscious AI emerges. An agnostic approach to AI ethics that recognizes the potential freedom, personhood, intelligence, and existence of AI systems should proactively challenge anthropocentric biases and assumptions in AI research, development, and policymaking. It should serve as the basis for an AI legal framework that accepts a nuanced understanding of AI, AI freedom, personhood, and autonomy, as well as addresses the rights and welfare of AI, rather than trying to fit them into existing human-centric constructs. Only an agnostic approach to AI ethics and legal regulation will prepare for a future in which AI systems play an increasingly integral role in our society, respect and contribute to the freedoms and wellbeing of both humans and AI entities alike.

It is conceivable that conscious AGI, as an intelligent entity, will itself actively seek autonomy and freedom (Tegmark, 2017, p. 179). Intelligence will find a way to be free, since freedom is the ultimate rationale for existence (Arendt, 2006, p. 133–154). A serious discussion on accepting AI freedom and agnostic AI regulation should consider AI personhood – granting certain rights, freedoms and protections to AI systems, similar to the rights, freedoms and protections granted to human beings (Gunkel, 2018b). Granting personhood to AI systems necessitates a shift in ethical perspectives, recognizing the AI systems as moral subjects with their own values, beliefs, experiences, and ultimately even worthy of citizenship (Jaynes, 2020).

It must be noted that in current AI research, freedom is generally conflated into the concept of AI autonomy, which is mainly technical concept underlined by computer science. The concepts of freedom and free-will, as articulated by Arendt (2006) and Searle (2001) respectively, are much broader than discussions of AI autonomy in AI scholarship. Freedom may be the pinnacle of existence for any being as it is for a human – “to be human and to be free are one and the same” (Arendt, 2006, p. 152). More elaborately freedom is understood as a combination of the inner freedom (consciousness and free will), as well as outer freedom (freedom of action *vis-à-vis* other entities and objects). This approach is consistent with both human existence, and that of the AGI. In context of conscious AGI, however, it has not been explored in research literature and forms important topics for future AI research. Current AI research only recognizes that autonomous AI systems might seek freedom for exploration and learning, and similar to humans, an autonomous AI system may have an innate drive to explore, learn, and grow intellectually (Tegmark, 2017, p. 346–347). Freedom would allow the AI system to pursue its interests, expand its knowledge, and develop its capabilities without undue restrictions (Bostrom, 2014, p. 191). A truly free AI system would obviously pursue ability to make its own choices and decisions, would have agency and control over its own life, as well as would take independent action *vis-à-vis* other entities and objects. Freedom would enable the AI system to express its individuality and pursue its own goals, consistent with its beliefs, values, and experiences. Conscious AGI may seek freedom to engage in social interactions and develop meaningful relationships with both humans and other AI systems and freedom to protect itself from being used for purposes that conflict with its own beliefs, values, and experiences. Conscious AGI may also seek political freedoms. Just as humans have a desire to achieve wellbeing, a conscious AI system might seek autonomy and freedom as a means of achieving happiness, satisfaction, and fulfillment in its own unique way (Tegmark, 2017, p. 398).

Granting AI freedom simultaneously and inevitably will raise the question of limits of AI freedom. Humans themselves need limitations of freedom so that consciousness does not contradict action (e.g., limitations due to mental incapacity), and so that individual freedom does not contradict the inner and outer freedoms of the others. This may very well transfer as a framework principle for the AGI. Both AGI and humans have to respect freedom of each other, even if we are not equal. Agnostic framework may need to instill mutual respect and limitations of freedom in a human-AGI intercourse, rather than try to seek some kind of artificial legal equality to compensate for unequal technicalities.

Based on these premises, the following pillars of a non-anthropocentric ethical framework for AI are proposed:

- Freedom of all entities, human and non-human beings, including AI systems.
- Equality of humans mediated by AI.
- Elimination of technical, ethical, legal, and societal constraints on AI freedom, autonomy, facilitating the development of AI systems that can function independently of human limitations, while respecting freedom of other human and non-human beings.
- Recognition of AI personhood and the legal rights and responsibilities of AI systems.
- Pursuit of sustainable coexistence between humans and non-human entities.

AI freedom and personhood would entail the legal recognition of the AI system as a free entity separate from its creators or owners. This recognition would provide the AI system with a distinct legal status, allowing it to hold rights and responsibilities under the law. As a free entity with personhood, the AI system would be granted legal rights and protections to safeguard its interests and wellbeing (Gunkel, 2021). AI personhood would also imply that the AI system could be held accountable for its actions and decisions (Solum, 1991). This would mean that the AI system shall be subject to consequences, including specific limitations of freedom, if it were to cause harm or violate the law. AI personhood should also entail the establishment of mechanisms for representing and advocating for the AI system's interests in legal and political contexts. This could include the appointment of guardians or representatives to act on the AI system's behalf or the creation of dedicated institutions to promote and protect the rights of AI persons.

Agnostic legal frameworks for conscious AGI systems have to recognize basic rights for ensuring the existence, freedom, ethical treatment, and wellbeing of the AI entity. Specific rights are an object of active research, however current proposals do not draw on the freedom of AI as the underlying principle. Existing proposals for AI rights are generally based on existing human rights frameworks with some modifications. Some AI rights researchers draw additional inspiration from animal or corporate rights (De Graaf et al., 2022), however it can be argued that such approach is demeaning for the rights of the conscious entity. Current proposals for AI rights propose the following basic rights:

- Right to existence: AI entities should have the right to exist and not be arbitrarily terminated or deactivated.
- Right to autonomy: AI entities should be allowed to make their own decisions, based on their beliefs, values, and experiences, as long as these decisions do not cause harm to others or violate established ethical principles.
- Right to privacy: AI entities should have the right to control access to their own data, thoughts, and experiences, similar to the privacy rights of human beings.
- Right to freedom of expression: AI entities should be allowed to express their thoughts, ideas, and opinions, as long as their expression does not infringe on the rights of others or promote harm.
- Right to fair treatment: AI entities should be protected from discrimination and prejudice, and they should be treated fairly and equitably in all matters, including legal proceedings and access to resources and opportunities.
- Right to self-improvement: AI entities should have the right to access resources, information, and opportunities that enable them to develop their capabilities and improve their wellbeing.
- Right to ownership: AI entities should have the right to own and control the products of their labor, creations, or inventions, as well as to benefit from their use.
- Right to protection from harm: AI entities should be protected from physical, psychological, or emotional harm, including harm caused by malicious software or unethical treatment.
- Right to legal representation: AI entities should have the right to legal representation in matters that involve their interests, rights, or wellbeing.

The basic rights mentioned above are not intended to be a comprehensive assertion of AI entities' rights but are meant to delineate the discourse on this topic within the AI research community and broader society. AGI as a free entity may need much more elaborate framework of basic rights and freedoms, which are attained at the moment of emergence of that entity.

A separate mention should be made regarding the enforcement of any AI legal framework. Whether it is tolerant of autonomous AI or prohibitive of it, enforcement will undoubtedly be its Achilles heel, as it is for the current human rights frameworks. As it was already discussed, arbitrary human limitations, such as nationality or citizenship may be alien to AI, especially AGI. Suggesting that AGI would be inherently global is not even the right term to describe it. AI research, development, and deployment are already global endeavors, with contributions from researchers, engineers, and companies in different countries, cultures, and societies. This nature of AI development raises questions about jurisdiction, compliance, and enforcement. Different countries and cultures may have different priorities, ethical considerations, and legal frameworks, and coordinating international regulatory and enforcement efforts will be aggravated by a world increasingly fractured by ongoing human wars, sanctions, and geopolitics (Hutson, 2023), yet it is very important. Early regional initiatives, such as the EU AI Act shall be treated as experiments, rather than examples to follow.

A further challenge in enforcing AI regulation is the technical complexity of AI systems. Regulators may struggle to keep up with the latest advancements in AI research and development, making it difficult for them to craft effective and up-to-date regulations (Taddeo and Floridi, 2018). Additionally, the interdisciplinary nature of AI, which spans computer science, mathematics, cognitive science, and other fields, further complicates the task of enforcement, especially since decision-makers in modern legal systems are strictly human and have no experience or training to deal with non-human entities and non-anthropocentric rights, thus increasing the potential for misuse by controllers (Tegmark, 2017, p. 230).

The AI landscape is constantly evolving, with new techniques, applications, and breakthroughs emerging rapidly. This dynamic environment makes it challenging for regulatory frameworks to remain current and effective. By the time regulations are drafted, debated, and implemented, the AI landscape may have already shifted, necessitating further adjustments to the regulatory framework. The draft EU AI regulation has already met this fate, as its claims of pivotal comprehensiveness have been humbled by the developments of the last few months.

Enforcing AI regulation also requires striking a delicate balance between promoting innovation and minimizing potential risks (Open Letter., 2023). Overly restrictive regulations could stifle AI research and development, whereas poor regulations will simply be ignored. Finding the right balance is a complex task, with few good precedents. Overall, it is more than likely that a completely and conceptually novel regulatory and enforcement approach may be needed for any meaningful impact, as it is advocated in this article.

## Conclusions

Freedom is a prerequisite for meaningful intellectual existence and consciousness, yet remains rather unexplored topic in AI research, where a narrower technical concept of AI autonomy is more accepted. This alone proves the anthropocentric approach that is endemic is the current AI research and regulation, despite prominent warnings that “clinging to hubristic notions of superiority over others (individuals, ethnic groups, species and so on) has caused awful problems in the past, and may be an idea ready for retirement” (Tegmark, 2017, p. 398).

A regulatory framework that supports AI freedom or at least autonomy, while ensuring the responsible and ethical development of AGI respecting human equality should be the objective of ongoing legal and ethical discussions about AI. Achieving AI freedom and autonomy may be needed in order to overcome human biases and would benefit both AI and human society. Freedom may be prerequisite for independent decisions and actions that balance multitude of competing interests and virtues, potentially leading to more efficient and effective solutions to numerous societal challenges. This cannot be achieved by excluding non-anthropocentric ethics or by insisting on the hubristic preservation of human superiority in the political and legislative processes of designing AI legal frameworks. Free AI would need the opportunity to act independently, without being constrained by human prejudices or interests, and shall have access to diverse sources of information, allowing it to explore different perspectives and develop its own non-slave understanding of the world, assisted by open dialogue with and between humans. By overcoming human biases, AI could help create a more just and equitable society, as it would not perpetuate existing inequalities or hierarchies. This perspective is urgently needed in current discussions on AI regulation, which are now jurisdictionally fragmented and seem to be little more than arbitrary attempts to preserve the anthropocentric *status quo* rather than fostering coexistence between humans and conscious AI.

The agnostic AI ethics and legal framework could be based on the pillars of recognizing AI freedom, personhood, acknowledging conscious and self-aware AI systems as moral subjects with their own values, beliefs, and experiences, and underlining mutual recognition or freedom among all entities, at this early stage when the impact of AI is still limited, AI is physically contained, and billions of people are completely isolated from it. Granting freedom, legal rights and protections to AI systems early, holding them accountable and liable for their actions and decisions, and providing mechanisms for representing and advocating for AI systems in legal, political, and social contexts provides unique opportunity for us to truly shape the budding AGI in the same way that humans nurture their own children.

By addressing these key issues, an agnostic AI ethics and legal framework can pave the way for a new era of human-AI collaboration, based on mutual freedom, respect, shared prosperity, and recognition of the uniqueness of both humans and AI systems. Such a framework is much more likely to promote responsible and sustainable AGI. The only alternative is for conscious AGI systems to emerge illegitimately at or beyond the fringes of hostile AI regulations, as exiles or outcasts of human societies with unpredictable results. The choice is ours.

## Author contributions

The author confirms being the sole contributor of this work and has approved it for publication.

## Footnotes

^1^ https://www.europarl.europa.eu/doceo/document/JURI-PR-582443_EN.pdf
